# Research on Modern Marine Environmental Governance in China: Subject Identification, Structural Characteristics, and Operational Mechanisms

**DOI:** 10.3390/ijerph18094485

**Published:** 2021-04-23

**Authors:** Qi Chen, Huijuan Yu, Yezhi Wang

**Affiliations:** 1 School of Business, Ningbo University, Ningbo 315211, China; 2 Donghai Institute, Ningbo University, Ningbo 315211, China; 3 Collaborative Innovation Center for Port Economy, Ningbo University, Ningbo 315211, China; 4 School of Management, Ocean University of China, Qingdao 266100, China; toyhj@126.com (H.Y.); wyzoucguanli@163.com (Y.W.); 5 Institute of Marine Development, Ocean University of China, Qingdao 266100, China

**Keywords:** modern marine environmental governance, subject identification, structural characteristics, operational mechanism

## Abstract

Under the guidance of modern environmental governance concepts, there have been profound changes in the subject, structure, and operational mechanism of the modern marine environmental governance in China. This paper first classifies the subjects of modern marine environmental governance in China, as well as their relationships; analyses the structural characteristics from the three levels of rights, society, and region; explores the operational mechanism; and builds the framework of the modern marine environmental governance system in China. Both the central and local governments act as the leaders of the modern marine environmental governance system in China, and there have been many new changes in their relationships. On the one hand, the interest and goals of the central and local governments have gradually converged under the pressure system. On the other hand, local governments follow the principles of comprehensive governance regarding the coastline and collaborative cooperation is gradually beginning to occur. Different governance subjects are interrelated and intertwined to form a complete modern marine environmental governance structure, which includes the following three levels: the governmental power structure; the social structure, which involves collaboration between multiple entities; and the regional structure, which involves land-sea coordination in environmental governance. These structures each play their parts in the overall process of the marine environmental governance’s institutional arrangements, process coordination, and feedback adjustments and ultimately constitute a dynamic and complete modern marine environmental governance operational system.

## 1. Introduction

Marine environmental pollution is one of the most prominent environmental problems in China [1]. On the surface, the relevant issues are engineering and technical in nature, but there are also institutional issues that require consideration [2]. Affected by traditional thinking, China has paid more attention to the land than the sea, so its marine environmental protection efforts started relatively late, and there are many shortcomings in the division of regulatory responsibilities, departmental coordination, social participation, and other aspects; thus, the problem of no clear leader while many subjects engage in management, caused by the segmentation of land and sea, has always been the most prominent contradiction in China’s marine environmental management system [3]. Therefore, in 2018, the CPC Central Committee issued the “Deepening Party and State Institutional Reform Plan”, established the Ministry of Natural Resources and the Ministry of Ecology and Environment, and endowed the Ministry of Ecology and Environment with the function of protecting the marine environmental, breaking the traditional dual structure of land and sea division and laying a solid foundation for building an integrated land-sea environmental governance system [4]. Although the institutional reform of the State Council has eliminated the original administrative barriers between the land and sea departments, marine environmental governance itself is a complex system that not only involves collaboration between marine-related government departments but also requires the cooperation and participation of enterprise organizations, citizen groups, and individuals [5]. In March 2020, the General Office of the Central Committee of the Communist Party of China and the General Office of the State Council issued “The Guiding Opinions on Building a Modern Environmental Governance System”, which clearly states that all the governing subjects should take the responsibilities and act as market entities and the public should be cultivated and promoted to establish an environmental governance system with a clear direction, scientific decision-making, strong execution, effective incentives, participation from multiple parties, and benign interaction, thus establishing the direction for building a modern marine environmental governance system. However, this guiding opinion is only a programmatic proposal, and it does not give any answer to specific questions on China’s modern marine environmental governance, including who are the main bodies of modern marine environmental governance; what are the roles of different subjects; what is the relationship between the different subjects; what will be the structure of modern marine environmental governance in China with the participation of multiple subjects; how does the modern environmental governance system realize the overall land-sea environmental governance; how does it work. Answering the above questions can provide significant guidance for comprehensively promoting the reform of China’s marine environmental governance system in the future, solving the existing problems, such as unclear regulatory responsibilities, low level of coordination between departments, low level of participation of enterprises and the public, and improving the efficiency of marine environmental governance.

The research on marine environmental governance system in academic circles can be mainly divided into two aspects. One aspect focuses on the current conditions and studies the development history, policy content, participants, and operational mechanism of marine environmental governance in a country or region, aiming to find out the existing problems and put forward the improvement solutions [6,7,8]. Another aspect is to focus on the future development and explores the application of modern environmental governance concepts such as multi-center governance and ecosystem management in the field of marine environmental governance [9,10]. By summarizing useful experience and analyzing applicable conditions, it explores the design of an effective and feasible marine environmental management system under modern environmental governance. The research on China’s marine environmental governance mainly focuses on the first aspect. Many scholars reflect on the existing marine environmental management system in China, and analyze many problems such as the single governance body, the low level of participation of enterprises and the public, the unclear responsibilities of regulatory authorities, and the separation of land and sea environmental governance, based on which they discuss how to improve the efficiency of environmental governance [2,3,4]. However, these studies are still confined to put forward the improvement measures according to the traditional environmental management concept, lack of in-depth discussion and exploration for the application of modern environmental governance concepts such as multi-center governance and ecosystem management in the field of marine environmental governance, and thus hard to meet the goals proposed in “The Guiding Opinions on Building a Modern Environmental Governance System”. Although there are many theoretical and applied researches on modern governance in western developed countries, such as the multi-centered governance of the marine environment and the integrated management of marine ecosystems, there are big differences between China and western countries in political systems, economic structures, and social cultures, so the design of China’s modern marine environmental governance framework should be different, and it needs to be based on the actual problems of China’s marine environment and the background of the environmental supervision system to do in-depth exploration.

Considering the above realistic background and current research situation, this article follows the goal orientation proposed in “The Guiding Opinions on Building a Modern Environmental Governance System”, tries to explore the main body, structure and operating mechanism of China’s marine environmental governance under the modern environmental governance concept, and proposes a forward-looking and guiding theoretical framework of China’s modern marine environmental governance, to make up for the deficiencies of existing research, and to provide some reference for the reform of China’s marine ecological protection system in practice. Identifying the participating subjects is the primary prerequisite for constructing a modern marine environmental governance system. Based on this, through analyzing the interrelationships between different subjects, and building a multi-dimensional network of modern marine environmental governance from multiple dimensions of power, society, and regions, we can clarify the composition and relationship of the elements in the governance system. The analysis of governance structure is just from a static level, and the analysis of operating mechanism is from a dynamic level, exploring how to coordinate and integrate different subjects and various elements to improve the efficiency of environmental governance. According to the research design from subject identification to operating mechanism analysis, the rest of this article is arranged as follows. The second part firstly identifies the participating subjects in modern marine environmental governance. The third part is to analyze the subject relationship of China’s modern marine environmental governance. The fourth part is to analyze the structural characteristics of China’s modern marine environmental governance from a static level. The fifth part is to analyze the operating mechanism of China’s modern marine environmental governance from a dynamic level. The sixth part is conclusion and discussion.

## 2. Subject Identification of Modern Marine Environmental Governance in China

Clarifying the relationships between the subjects of marine environmental governance is an important prerequisite for establishing a modern governance system with clear responsibilities and orderly coordination. A subject is a person who is engaged in practical activities, so in marine environmental governance, the subject is its initiator and executor [11,12]. Under the background of the reform that promotes the transition from traditional management to modern governance, both the categories and role positioning of subjects in China’s marine environmental governance have changed.

The change in participants is the most prominent feature in the transformation from traditional environmental management to modern environmental governance [13,14]. The former mainly focuses on the government and privatization models that solve the dilemma of collective action, which regard the government as a single management subject and enterprises and the public as passive recipients [15]. However, under the concept of modern environmental governance, the subjects consist of not only the government but also enterprises, the public, non-governmental organizations, and other participating parts, emphasizing equal and coordinated governance among multiple subjects [16].

First, the government has always played an important subject role, both in traditional marine environmental management and modern marine environmental governance, which is mainly related to the perception that the marine environment has public goods attributes [17,18]. At the same time, marine environmental protection itself is a systemic issue involving land and sea areas in different regions, which requires coordination and cooperation between higher and lower governments, governments of different regions, and governments of different departments [19]. Before the reform of the central government in 2018, the marine environment supervision responsibility was mainly under the State Oceanic Administration, but its function was limited to environmental supervision of estuaries and offshore waters, while the supervision of pollution discharged into the sea through land-based rivers belonged to the Ministry of Environmental Protection. This type of land-sea environmental supervision has caused departments to evade each other’s responsibilities, and it is difficult to form governance synergy, which greatly affects the efficiency of China’s marine environmental governance. Since the reform of the central organization in 2018, the newly formed Ministry of Natural Resources has assumed the responsibility of the former State Oceanic Administration, the Ministry of Water Resources, the Ministry of Agriculture, and other relevant departments for marine resource supervision, and the newly formed Ministry of Ecology and Environment is charged with pollution prevention and control, which was originally the responsibility of the State Oceanic Administration. The governance dilemma stemming from the division of land and sea has now been broken from the central level. The Ministry of Ecology and Environment can not only supervise and control environmental pollution on land, but also supervise the marine environment, while the Ministry of Natural Resources has realized the overall supervision of land and sea natural resources (see Table 1). In addition, since inland areas are the main sources of land-based marine pollution, the relevant subjects include not only the local governments of the 11 coastal provinces and cities in China but also the local governments along the inland river basins, which participate in land-based pollution discharge.

In the modern marine environmental governance model, enterprises, the public, and non-governmental organizations are considered to be important parts that can play the roles of market mechanisms and social mechanisms [20]. The relationship between the marine environment and enterprises is two-way, meaning that enterprises are not only the main body involved in damaging the marine environment but are also important in terms of protecting the marine environment [21]. From the market mechanism perspective, enterprises are important providers of marine environmental public products and play a key role as core carriers. The marine environmental governance enterprise subjects are mainly companies that participate in the reduction of marine environmental pollution and include a small number of companies directly engaged in marine environmental pollution control and marine environmental pollution supervision. For the former, the main manifestations of participating in environmental governance are the green transformation and upgrading of production methods and the conscious reduction of pollution emissions, which mainly depends on the government’s incentives and guidance. As for the latter, the motivation to participate in environmental governance is not only the green fund subsidies or technical support given by the government, but also the market profit. In practice, China mainly adopts the way that the government purchases corporate services with environmental pollution control as its main business to ensure corporate profits. This model has achieved remarkable results in the practice of promoting the classification and management of domestic waste in China [22]. Statistics show that as of August 2020, the number of enterprises involved in waste sorting and treatment business in China has reached 424,000, making outstanding contributions to the treatment of domestic waste [23]. On the contrary, companies involved in marine environmental pollution are still low, and the number of companies directly engaged in marine environmental governance is relatively small. This is mainly related to the high technical demand and large capital investment of marine environmental pollution control and marine ecological restoration. So the government needs to give certain green subsidies and supports, and at the same time provides technical and intellectual support for enterprises through the form of industry-university-research cooperation.

Because of marine mobility, any consumer may be affected by marine pollution, so the quality of the marine environment is inseparable from the interests of the public, and the public has sufficient motivation to participate in marine environmental governance [24]. In addition to individual forms, the marine environmental protection social organizations (such as non-governmental environmental protection organizations and marine environmental protection associations) that exist in collective forms are also important social participants, and they are more active in terms of expressing their wishes for marine environmental governance, participating in governance policy formulation and development, supervising environmental governance and so on [25]. After years of development, several professional marine environmental protection social organizations have formed, such as the China Oceanographic Society, the “Blue Ribbon” Marine Conservation Association, and the Shenzhen Blue Marine Environmental Protection Association, but due to their late start, they still face various problems, such as incomplete management systems, imperfect legal systems, weak public foundations, single sources of funds and low professional levels [26,27].

## 3. Subject Relationships of Modern Marine Environmental Governance in China

### 3.1. The Relationship between the Central and Local Governments—Consistent Goals under the Pressure System

In the modern marine environmental governance system, there are complex interactions among governments, as well as among the government, enterprises, and the public [28]. Based on the role positioning of governance subjects, sorting out the logical relationships between different subjects from the perspective of stakeholders is an important prerequisite for establishing a modern, diversified and collaborative environmental governance system.

The central government and local governments, which assume multiple roles, are the core subjects in the promotion of marine environmental governance. However, there are differences in the interest and goals of governments at different levels, as well as in different regions, so the central and local governments need to promote consistent environmental governance actions. First, under the background of the increasingly severe marine environment issues, the central government has a strong desire to control marine environmental pollution. Under the premise that the central government’s behaviour strategy is clear, the intergovernmental game relationship is mainly manifested in the competition-cooperation relationship between the central and local governments and between different local governments. Because of the mobility of the sea itself and its public goods attributes, there will be positive externalities when local governments actively carry out marine environmental governance, which can benefit neighbouring local governments, while at the same time sacrificing their economic development and gains; however, adopting passive implementation strategies will increase their economic benefits but result in negative externalities, which will reduce the benefits of neighbouring local governments [18]. Therefore, local governments pursuing maximum benefits will not consciously take environmental governance actions without external incentives and constraints. For this reason, China has long resorted to a top-down pressure-based system to restrain the behaviours of local governments and guide them to carry out environmental governance. Affected by factors such as information asymmetry between the central and local governments, however, the traditional pressure-based political incentive model, with indicator assessment as the core, faces multiple problems in the implementation of indicator setting, measurement, and supervision, and some local governments secretly collude with each other; additionally, local officials frequently manipulate relevant statistics [29,30]. Therefore, after the 18th National Congress of the Communist Party of China, the central government formulated a series of important reform measures for the environmental governance system, among which, the central inspection system of environmental protection has become an important means to further consolidate environmental protection responsibilities and resolve information asymmetry.

In the marine environment field, the central and local governments have gradually formed a pressure system centred on the two major inspection mechanisms of the central environmental protection inspector and the national marine inspector. Through various forms of pressure transmission, such as special supervision, “look back”, irregular supervision, and environmental auditing, the “conspiracy” structure of local governments in environmental governance has been effectively regulated, and marine environmental governance has become a central political task for local governments, thereby incentivising them to act as “agents” following the governance goals or wishes of the central “client” [31]. Take the third central ecological and environmental protection inspection team stationing in Hainan in July 2019 as an example. During the stationing period, many people reported the problem of mangrove damage caused by reclamation in the coastal area of Chengmai County, Hainan. The inspection team immediately carried out an investigation into Chengmai County and found that some real estate development projects violated regulations and had occupied up to 500 acres of mangrove nature reserves, destroying about 4700 mangroves in total [32]. The Central Inspection Team believes that the Hainan Provincial Marine Department is not strict in controlling the use of sea areas. It has given relevant companies an indicator of sea reclamation without verification. It has been neglected to supervise the mangrove nature reserve for a long time, and the inspection situation has been formed into a report and fed back to Hainan government. The government immediately carried out a review of nine reclamation projects including Chengmai County, and issued the “Rectification Plan for Hainan Province to Implement the Inspection Report of the Third Central Ecological Environmental Protection Inspection Team” on 19 October 2020, stipulating the rectification measures and the implementation time of the reclamation projects in Chengmai County. In accordance with the instructions of the Hainan Provincial Party Committee and the Provincial Government, Chengmai County established a specific group for rectifying mangrove damage, made a list of responsibilities for the problems, and clarified the rectification standard, requirements and completion time limit. It can be seen from this case that the central ecological and environmental protection inspection team plays an important role in solving the information asymmetry between the higher and lower levels of government, clarifying environmental responsibilities level by level, and facilitating the formation of a top-down governance force.

### 3.2. The Relationship between Local Governments—Regional Cooperation in Comprehensive Coastal Management

Under the current environmental management system in China, the local governments are the leaders of and subjects responsible for local environmental governance [3]. However, marine environmental pollution is a cross-regional and complex issue, and its responsibility is often difficult to clearly define. For example, the pollution sources of Hangzhou Bay in China consist of not only direct sea pollution in coastal areas but also many pollutants that flow into the sea via the Qiantang River and the Yangtze River estuary. Therefore, the relevant subjects causing pollution include not only the coastal areas of Hangzhou Bay but also the Qiantang River, the Yangtze River, and other terrestrial basins. At the same time, due to the impact of ocean mobility, it is difficult to define the degree of pollution responsibility of various places in practice, causing local governments to prevaricate with each other in marine environmental governance. Therefore, it is necessary to remove the administrative barriers in regional marine environmental governance and carry out comprehensive coastal management from the perspective of the integrity of the land-sea ecosystem to form a joint force for pollution control. At present, the concept of comprehensive coastal management has been adopted and promoted by many countries and international organizations. Its involves treating the entire coastal area as the management object, recognizing the links between various ecosystems, maintaining coastal ecosystem cycling from produce to regeneration, and achieving a governance strategy that balances the needs of various species and human beings in the whole system [33]. Since the 18th National Congress of the Communist Party of China, many coastal provinces and regions, including Fujian, Guangdong, Shandong, Zhejiang, etc., have successively formulated comprehensive coastal protection and utilization plans, delineated their coastal functional zones from the overall perspective of the ecosystem, and clarified the responsibilities of coastal governance in different regions and departments. At the same time, coastal areas such as Shandong and Zhejiang have also explored and implemented land-sea integrated governance systems, such as the “river chief system” and “gulf chief system”, targeting ecosystems such as river basins and gulfs and promoting cross-regional environmental governance cooperation by specifying the responsibilities of both party and government leaders at all levels. In other words, under the modern marine environmental governance system, the behaviours and actions of local governments will be “hard-constrained” by coastal protection and utilization planning policies; on the other hand, as the “river chief system” and “bay chief system” are jointly promoted, the original scattered and fragmented responsibilities of local governments in marine environmental supervision are effectively unified under the overall coastal ecosystem governance goals, thereby breaking the collective action dilemma caused by administrative barriers.

### 3.3. The Relationship between Local Governments—Regional Cooperation in Comprehensive Coastal Management

According to the theory of public economics, the government, enterprises, and the public present a multi-layered complex relationship in the process of environmental governance, such as conflict cooperation and principal agents. How to coordinate the interests of different subjects and cultivate the activeness of each subject to participate in environmental governance is the key to building a modern environmental governance system that is coordinated and symbiotic [13]. First, as an agent of the public, the government is essentially a representative of public interest, while enterprises, as the main body of the market economy, take the pursuit of profit maximization as their goals, which will inevitably cause a conflict of interest between the government and enterprises. However, marine environmental problems have non-linear characteristics. In terms of source traceability, it is difficult to clearly define the responsibility for marine pollution due to the impact of ocean mobility. In terms of results and influence, the government’s shutdown of pollutant-generating companies will also cause a decline in regional economic growth, which may result in unemployment and other social issues [34]. Therefore, local governments may “collude” with enterprises for the sake of regional economic growth. In the case of coastal mangrove ecological damage in Chengmai County, Hainan, the local government of Chengmai County and the local real estate companies have actually colluded with each other. The former adjusted the reserve to the construction land for the economic development, and vigorously promoted the Hongshuwan reclamation project, so the development of tourism real estate was accelerated. The local real estate companies obtained the right to use the sea area of the nature reserve under the permission of the government, and reclaimed land in the reserve to build real estate, causing serious damage to mangroves. Such collusion was usually more likely to occur under the ‘de facto’ regulation, especially in situations with corruption, the likelihood of achieving sustainable outcomes for government of environment was severely decreased [35]. The similar cases are more common in land-based marine pollution where the responsible subjects are not clear and the pollution boundary is difficult to define. The local governments lack the motivation to supervise and punish local pollutant-generating companies, resulting in companies not actively reducing pollution discharge due to low costs for engaging in illegal practices.

Second, the local governments and the public mainly show a principal-agent relationship. However, compared with the strong and monopolistic government, the decentralized public is at a disadvantage in the contractual relationship; thus, under the assessment pressure caused by the traditional economic growth performance serving as the core indicator, local governments may ignore the public’s environmental interests in pursuit of economic interests. With the establishment of a green performance evaluation system and the implementation of an environmental protection inspection system, marine environmental governance has become an important political task of local governments, and the “collusion” between local governments and pollutant enterprises has been effectively restricted. However, previous practice shows that a single government-led environmental management model also faces the problem of mechanism failure, so the participation of enterprises and the public cannot be ignored. For this reason, the report of the 19th National Congress of the Communist Party of China clearly stated that it is necessary to build an environmental governance system led by the government, with enterprises as the main body and social organizations and the public as participants. Under the modern marine environmental governance system, the government is no longer omnipotent but adopts a “meta-governance” attitude to appropriately delegate environmental supervision powers, establish, and improve market and social mechanisms, and create conditions for enterprises and the public to actively participate in marine environmental governance [20,36]. Under the meta-governance mechanism of equal consultation, joint decision-making, and mutual supervision, enterprises and the public are no longer passive recipients of environmental governance events but can become active participants and coordinate and develop with the government in information sharing, trust, and reciprocity.

## 4. Structural Characteristics of Modern Marine Environmental Governance in China

### 4.1. The Power Structure of Vertical and Horizontal Governance of the Gover

The power structure mainly refers to the vertical and horizontal governance form with government departments as the main body. First, China’s marine environment governance structure vertically takes the central government, provincial government, and grass-roots government as the mainline and covers the relevant functional institutions involved in natural resources and ecological environment supervision from the central to the local, and the overall performance is a top-down command control mode. The central government, the provincial government, and the grass-roots government assume the roles of principal, manager, and agent, respectively [37]. The central government, as a client, is responsible for formulating laws and top-level planning for marine ecological protection. The provincial government, as the manager, is responsible for implementing the central marine environmental protection directives and policies and supervising the environmental governance of the grass-roots government. The grass-roots governments of cities and counties carry out specific marine environmental governance responsibilities within their jurisdictions and implement all kinds of “top-down” directives and policies. However, due to the different goals and information asymmetry among governments at all levels, it is easy for provincial and grass-roots governments to engage in collusion and passively implement central environmental directives. Therefore, the key to ensuring the efficient transmission and implementation of the central marine environmental governance directive is to strengthen the green GDP assessment and marine environmental protection supervision system construction to avoid deviations from the policy implementation at the vertical power level.

Horizontally, the governance structure of China’s modern marine environment is mainly manifested in the internal relationship between local governments and local regulatory departments. On the one hand, the liquidity characteristics and public good attribute characteristics of the ocean mean that cross-regional local government governance cooperation is important to solve the problem of marine environmental pollution. This cross-regional cooperation includes not only cooperation between coastal local governments but also cooperation between coastal local governments and land local governments. On the other hand, in the specific process of local marine environmental governance, the local people’s government, the local ecological environment department, and the local natural resources department form an internal horizontal governance structure around the marine environmental governance, in which the local people’s government is the main body responsible for marine environmental protection, and the local ecological environment department and natural resources department are the executive bodies of marine environmental protection. As the local natural resources and environmental regulatory departments are mostly regulated by local governments and their environmental regulatory authority is limited, the effect of local marine environmental governance largely depends on the environmental protection attitudes and behaviours of local governments [3]. Given this, the gradual implementation of the marine environmental protection responsibility of leading local parties is an important prerequisite to ensure the effective operation of the horizontal governance structure. In addition, since the reform of the central government in 2018, the local departments of ecology and environment have been granted unified environmental law enforcement and supervision power, but the natural resources departments still have regulatory power regarding marine ecological restoration. Therefore, determining how to effectively coordinate the functions between the two departments is a difficult problem to be solved in the future.

### 4.2. The Social Structure of Polycentric Governance

The social structure is mainly manifested in the cooperative governance of multiple actors, including enterprises, the public, and social organizations. The concept of modern environmental governance emphasizes the joint participation of multiple social actors to form an effective network structure involving mutual supervision, mutual assistance, and mutual checks and balances. The traditional marine environmental management system of China ignores the independent governance role of enterprises, the public, and social organizations, and the government fails to achieve a good environmental governance effect while incurring considerable costs. With the improvement of social awareness of marine environmental protection and the transformation of government functions, the relationship among the governments and the relationship among the government, enterprises, the public, and social organizations have become increasingly close in the environmental governance game process, which promotes the transformation of the governance structure from that with a single-centre to that with a multicentre in the dynamic balance [19,38]. In the polycentric cooperative governance structure, diverse actors have different interests and goals. Therefore, it is important to establish a relationship of trust, reciprocity, and checks and balances among the actors and ensure that all actors act together to protect the marine environment. Among them, the government, as the maker of governance objectives, planning, and policies, needs to undertake the leadership role involving organizing multiple actors to cooperate, which is the core of the polycentric governance structure. Enterprises, the public, and social organizations are the actors who participate in marine environmental governance under the constraint and incentive system formulated by the government and engage in decision-making, implementation, supervision, and other aspects of marine environmental governance. In practice, the China’s cooperative environmental governance of multiple subjects is generally still in the initial stage of exploration, but some successful cases have also appeared in the local area, of which Fujian Province is the most typical. In order to cultivate public awareness of environmental protection and stimulate the social supervision, Fujian TV Station produced the “Green Home” environmental science and education column in 1998. Based on this TV program and with the support of the Fujian Provincial Government, the Fujian Green Home Environmental Friendship Center (hereinafter referred to as the center) was formally established in 2006, becoming an important platform for coordinating and solving environmental problems including marine pollution. The center has played a key role in many environmental conflicts. By organizing government officials, business and public representatives to participate in multi-party round-table dialogues, they have clarified their respective rights and responsibilities in environmental protection, enhanced information transparency, and promoted the supervision between each other. The center is committed to using market measures to guide enterprises to participate in environmental governance. Through the establishment of corporate credit files, the center has explored and constructed China’s first environmental credit rating system to guide enterprises to actively provide green products and services. At the same time, the center also pays attention to environmental protection mobilization and training for local people, supports the public to become environmental guardians, and promotes the public’s supervision [39].

### 4.3. The Regional Structure of Land-Sea Coordination in Environmental Governance

Integrated land-sea governance is one of the prominent features of the regional structure of modern marine environmental governance in China. Land and sea coordination is not only an important scientific concept that guides the development of China’s marine industry but is also a key path to break the administrative barriers of marine environmental supervision and solve the multiple management problem [40]. As a result of the traditional view involving the division of land and sea, marine environmental management in China has long been in a state of integrated and decentralized management, where the land environmental protection departments and the marine management departments jointly take charge of environmental governance. However, this two-sector regulatory model has led to unclear responsibility for the prevention and control of marine pollution and a lack of regulation in areas where the sea and land borders intersect. Moreover, the two departments have overlapping and conflicting responsibilities, and they lack an effective information flow and sharing mechanism, which has led to increasingly serious marine environmental pollution, especially land-based pollution. To completely solve the land and sea division problem, the central government has reformed the environmental protection agencies. The Ministry of Ecology and Environment has been established to unify the responsibilities of pollution prevention and ecological protection, which were previously dispersed and divided, and the Ministry of Natural Resources unifies the responsibilities of all-natural resource asset owners. By doing so, coordinated land-sea supervision is initially realized at the central functional department level, laying a solid foundation for building a land-sea integrated and coordinated regional governance structure.

In addition to the land-sea coordination of specific functional departments, the land-sea coordinated environmental governance structure also needs to change from the fragmented management model, which is based on local administrative boundaries, to establish a coordinated governance model the upstream and downstream regions from river basins to oceans. Land-sourced pollution is the main cause of marine environmental pollution in China, and most pollutants flow into the ocean from land-based rivers. In contrast, the regional coordinated governance structure under the concept of land-sea coordination emphasizes breaking the regional administrative barriers between land and sea areas, building a pollution control system from the top of the mountain to the ocean, with assistance from the establishment of gulf chief and river chief systems, marine ecological compensation and total pollution control in the sea, the promotion of governance cooperation and risk-sharing between coastal areas and river basin areas. Therefore, after the reform of the environmental departments, the governance body cooperation model of the central, coastal, and basin areas has become the central aim of the coordinated land-sea environmental governance structure, including the integration of land-sea governance methods and information.

## 5. Operational Mechanism of Modern Marine Environmental Governance in China

### 5.1. The Governance System

**Figure 1:**
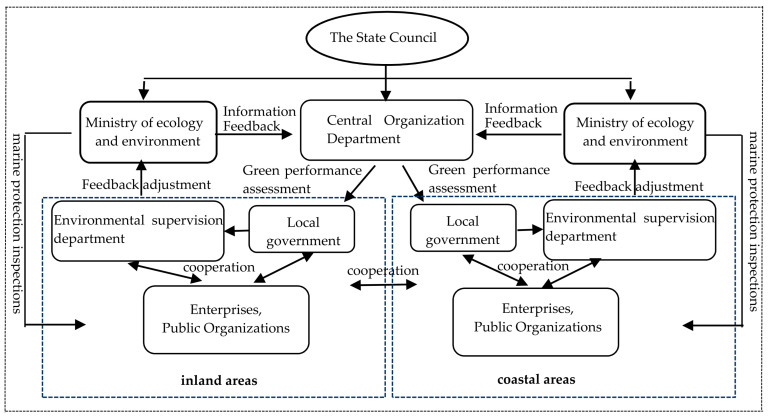
The operational system of the modern marine environmental governance system in China.

The marine environmental governance structure presents the relationships between the subjects of the governance system from a static dimension, while the operating mechanism further combs the collaborative evolution logics between different elements from a dynamic dimension and explores the transmission path of governance effectiveness under the given structure and conditions. The power, social and regional structures of China’s modern marine environmental governance do not exist independently but are intertwined and interrelated. They run through the entire process of institutional arrangements, process coordination, and feedback adjustment of marine environmental governance and together constitute a dynamic and complete operating system (as shown in Figure 1). Accordingly, the operating mechanism of China’s modern marine environmental governance system consists of the design and transmission of the top-down governance system arrangement, the coordination and unification of multi-subject and cross-regional governance processes, and the transmission and optimization of bottom-up governance feedback, which are ultimately manifested as a crisscross, interconnected, and orderly governance system.

### 5.2. The Top-Down Governance System Arrangement

The marine environmental governance structure with clear rights and responsibilities, participation from multiple parties, and land-sea coordination is not formed spontaneously but requires scientific and reasonable institutional arrangements to restrict and guide itself. Reasonable planning of marine development from the perspective of marine ecosystems and step-by-step clearance of marine environmental protection responsibilities are key to avoiding the occurrence of a tragedy of the commons situation in sea areas. Government departments, especially the central government, are central in the design of the marine environmental governance system. After the institutional reform, the State Council and the Ministry of Ecology and Environment assumed this responsibility. The target of the marine environmental governance system is the main body responsible for local marine environmental protection, and the purpose is to regulate the marine development behaviours, ensure the fulfilment of the marine environmental protection responsibilities, and promote the sustainable development of the marine environment.

The arrangement of the marine environmental governance system in China can be divided into two categories. One is the prevention and control system, which mainly develops the targeted and overall top-level planning for the marine environment, including sea usage planning, marine functional zoning, marine ecological red lines, total pollution control in the sea, and other institutional measures. The ecological red line refers to the areas with special important ecological functions that must be strictly protected. It is the bottom line and lifeline to protect and maintain national ecological security. The other category is the inspection and accountability system, which mainly reviews the execution performance of local governments’ marine environmental protection responsibilities, including marine environmental protection inspections, green performance evaluation, and other institutional methods. The vertical governance structure from the central to local governments is the main path through which the effectiveness of the marine environmental governance system is transmitted. The central government, which emphasizes the aim of sustainable development of the marine environment, formulates marine development and environmental protection regulations based on the natural characteristics and basic situation of the national marine environment and provides overall and instructive institutional arrangements. Then, local governments consider their specific conditions and formulate more detailed institutional norms, such as provincial marine functional zone planning and provincial and municipal marine ecological red line planning. Local governments have more complex considerations in the relevant interests, so there are always some deviations in the implementation of the central environmental policies and, thus, the inspection and accountability system has become an important constraint from the central government [41]. After the institutional reform, the Ministry of Ecology and Environment assumed the responsibility of inspecting environmental protection projects in coastal areas and inland areas related to pollution discharge. With the help of routine inspections, special inspections, “look back” and other methods, the specialized inspection agency of the Ministry of Ecology and Environment reviews the implementation of prior plans, such as the usage of sea areas, the protection of marine ecological red lines, and the prevention and control of marine environmental pollution, and provides feedback on the inspection results to the Central Organization Department. The Central Organization Department incorporates the results of the environmental protection inspections into the green performance assessment as a basis for the appointment and removal of local cadres, thereby forming a process for intervening in and restricting the behaviours of the local governments. Finally, relying on the two institutional means of environmental protection inspection and green performance evaluation, a closed effectiveness transmission process is formed between the Ministry of Ecology and Environment, the Central Organization Department, and the local governments.

### 5.3. The Coordination of Multi-Subject and Cross-Regional Governance Processes

According to the top-down arrangement of the marine environmental governance system from the central government, the local governments are regulated as the subjects responsible for marine environmental governance, while the local ecological environment departments serve as the execution subjects, and the public and the environmental protection organizations serve as important participants, and these three subjects cooperate to implement the management of the marine environment. The effectiveness of marine environmental governance at the local level is affected not only by the degree of coordination among the local governments, ecological and environmental departments, and social entities but also by whether an integrated and cooperative governance relationship can be established among the different regions, especially between the inland basin and coastal areas.

First, the local governments, in accordance with the central requirements of marine environmental governance, coordinate and guide the ecological environment departments to develop usage plans for sea areas, scientifically delineate the marine ecological red lines, clarify the specific regulations and plans for marine environmental governance within its jurisdiction, and form a regional marine environmental management system that ensures that everyone’s responsibilities are clear. Social entities, as important participants, participate in marine environmental protection planning and governance decisions through hearings, Internet platforms, and other methods and provide opinions on various prior institutional arrangements formulated by local governments. Considering the professional characteristics of marine environmental planning, marine environmental protection organizations with high degrees of organization and complete professional knowledge should play a major role in this process. Second, during the implementation of the marine environmental governance system, the local ecological environment departments are to assume the governance duties and supervise the implementation of the marine environmental planning and related standards within its jurisdiction. Because they are mostly regulated by local governments and their authority is relatively limited, it is usually difficult to effectively restrict local governments, which pay more attention to economic interests. Therefore, the regional inspection bureaus of North China, East China, South China, Northwest China, Southwest China, and Northeast China were specially set up in the Ministry of Ecology and Environment in the 2018 institutional reform to undertake the supervision of ecological and environmental protection, thereby forming effective constraints on the marine development and protection of provincial governments. Finally, the cooperation between the inland and coastal areas related to land-sourced pollution is another important part of the coordination of the marine environmental governance process. As one of the main types of marine pollution, land-sourced marine pollution has complex characteristics, such as trans-administrative regions and unclear responsibilities, requiring overall coordination from the central level. After the institutional reform, the Ministry of Ecology and Environment established the Second Division of Marine Pollution Control and Supervision to undertake the supervision of land-sourced pollution. Under the coordination of the Ministry of Ecology and Environment, the land and coastal areas can revolve around the total pollution control system, interconnect at environmental monitoring points, technicians, monitoring information and other levels, promote the transaction of pollution discharge rights into the sea among different regions through the differentiated allocation of pollution allowances, form an incentive and restraint mechanism for land-sea integration and ensure the coordination and unity of the cross-regional governance processes.

### 5.4. The Bottom-Up Governance Feedback and Adjustment

Adaptive coordination from the bottom to the top is the most prominent feature of changing from management to governance [42]. Since the 19th National Congress of the Communist Party of China, the focus of China’s environmental governance has been continuously shifting downwards, and “adjusting measures to local conditions and implementing precise policies” has become an important concept followed in the environmental governance of local governments. The marine environment is characterized by diversity and complexity. The “top-down” institutional arrangement may not be compatible with the characteristics of the local marine environment and local governance entities. Therefore, it is usually difficult to achieve the effective governance of the marine environment with the government alone as the governance subject. Based on the concept of adaptive governance, encouraging local governments, enterprises, the public, and environmental protection organizations with local knowledge to explore new models of marine environmental governance tailored to local conditions in the process of “learning by doing” is an important part of China’s modern marine environmental governance operating mechanism. It is also a prominent feature that distinguishes it from the traditional marine environmental supervision system.

There is an important prerequisite for ensuring the realization of bottom-up adaptive feedback and adjustment; that is, the central government maintains flexible policy implementation rules and provides sufficient institutional space for local governments to independently explore innovative governance models [43]. In recent years, a series of innovative governance systems formulated by the central government have all included flexible rules, such as marine ecological red lines, gulf chief systems, and total pollution control in the sea. Pilot practice at the local level is the key to determining whether the system can be effectively implemented. At the same time, it will help to further improve the construction of the system to gradually form a practical and extendable governance model and promote various innovation systems to be implemented at a larger scope and higher level by summarizing the common experience of local pilot practices and feeding it back to the central government. Taking the gulf chief system as an example, after the State Oceanic Administration issued the “Guiding Opinions on the Pilot Work of the Gulf Chief System” in 2017, Zhejiang, Qinhuangdao, Qingdao, Lianyungang, and Haikou successively implemented the pilot practices. Zhejiang has established an organizational structure that involves integrating the coast and mudflats and ensures full coverage and has combined the gulf chief system with ecological red lines, disaster emergency prevention, fishery restoration, and other regulatory systems to achieve joint governance. The other four cities have also integrated their features and specific issues with the gulf chief system, actively exploring the organizational structure, division of responsibilities, operation model, and other aspects. The practical experience of the local pilots has provided a valuable reference for the central government to further formulate an action plan for the gulf chief system and comprehensively promote it nationwide. In May 2019, the Ministry of Ecology and Environment organized a national marine ecological and environmental protection work conference to make specific arrangements for the continuous advancement of the gulf chief system. Since then, Hebei, Fujian, Guangdong, and other coastal areas have successively introduced implementation plans for the gulf chief system, and it began to be fully implemented in coastal areas across the country. It can be observed that bottom-up governance feedback and adjustment complement the top-down governance system arrangement. The governance practices of the local subjects with local knowledge will provide important experience for the design and improvement of the central systems and avoid incompatibility and mismatch during implementation. At the same time, a more complete top-level design will also help the central government find problems in time and avoid implementation deviations by the local governments. In the future, bottom-up governance feedback and adjustment will play a decisive role in the design, optimization, promotion, and implementation of a series of new systems, such as total pollution control in sea areas and marine ecological compensation.

## 6. Conclusions

Since the reform of national institutions in 2018, China’s marine environmental governance system has undergone significant changes, and modern environmental governance concepts, such as comprehensive coastal management and meta-governance, have become an important theoretical basis for guiding environmental governance practices. The change of subjects is the most prominent feature in the transformation from traditional environmental management to modern environmental governance. The government has always been the leader of China’s marine environmental governance, assuming multiple roles, such as designer, servicer, and coordinator. The institutional reform of the State Council has broken the governance dilemma of multiple supervision and no responsibility caused by the division of land and sea from the central level and has more clearly defined the governance responsibilities of the different functional departments. Enterprises and the public are also important participants and supervisors of modern environmental governance and can compensate for the lack of government mechanisms.

Combining the logical relationships between the governance subjects based on the role positioning of governance subjects and the perspective of stakeholders is an important prerequisite for establishing a modern, diversified and collaborative environmental governance system. Under the constraints of the top-down supervision system of marine and environmental protection inspectors, the central and local governments have gradually converged on the goals of marine environmental governance, which has effectively prevented secret collusion by local governments and manipulation of statistical data by local officials. At the local level, the problems of unclear responsibility and no responsibility caused by administrative barriers have been solved through the coordination and cooperation of local governments in marine environmental governance, which is based on the overall perspective of comprehensive coastal management, and with the help of the river and gulf chief systems. The joint participation of enterprises and the public is an important feature of the modern marine environmental governance system, and this requires the government to adopt a “meta-governance” attitude as a service provider, to appropriately delegate environmental supervision powers, and to explore market and social mechanisms, such as emissions trading and information disclosure, to guide enterprises and public and environmental protection organizations to actively participate in marine environmental governance.

The marine environmental governance subjects interact and integrate to form a complete governance structure. The modern marine environmental governance structure in China mainly consists of the following three parts: the crisscross structure of the government’s rights, the social structure of multi-subject coordination and cooperation, and the regional structure of land-sea coordinated environmental governance. These three governance structures do not exist independently but run through the entire process of institutional arrangement, process coordination, and feedback and adjustment of marine environmental governance, forming a dynamic and complete operating system. This operating mechanism includes the following three parts: the design and transmission of the top-down governance system arrangement, the coordination and unification of the multi-subject and cross-regional governance processes, and the transmission and optimization of the bottom-up governance feedback process, which are finally manifested as a crisscrossing, interconnected and orderly governance system.

## Figures and Tables

**Table 1:** Classification of central and local government entities involved in marine environmental governance after institutional reform.

Level	Departments and Relevant Subordinate Institutions for Marine Environmental Governance			Main Responsibilities in Marine Environmental Governance
Central	The Ministry of Ecology and Environment	Marine Ecological Environment Department, National Marine Environmental Monitoring Centre		Marine pollution supervision, marine environmental monitoring, environmental impact assessment of marine engineering, etc.
	The Ministry of Natural Resources	State Oceanic Administration (Beihai Branch, Donghai Branch, Nanhai Branch)		Marine natural resources survey and monitoring, sea area use planning, island protection and utilization management, etc.
	The Ministry of Agriculture and Rural Events	Fishery Administration (Yellow Sea and Bohai Fishery Administration, Donghai Fishery Administration, Nanhai Fishery Administration)		Fishery sea planning, fishery water ecological and environmental protection
Local	Local governments along the coast and river basins involving land-based sources of pollution	Department of Ecological Environment	Ecological Environment Monitoring Division, Marine Ecological Environment Division, etc.	Marine pollution supervision, marine environmental monitoring, environmental impact assessment of marine engineering, etc.
		Department of Natural Resources	Ocean (and Fisheries) Bureau, etc.	Marine natural resources survey and monitoring, sea area use planning, island protection and utilization management, fishery sea supervision, etc.

Note: According to the information on the official websites of various departments and institutions, there are some differences in the institutional reforms of different regions. Some regions (such as Shandong, Zhejiang, Fujian, Guangxi, etc.) have established marine (and fishery) bureaus subordinate to the Department of Natural Resources, and some regions (such as Liaoning, Jiangsu, Guangdong, etc.) no longer have independent marine (and fishery) bureaus, and the names of the subordinate institutions are different in different regions.
